# Community Health Nurses’ Perspective on the Introduced Rational Drug Use Policy in Primary Care Settings in Thailand: A Descriptive Qualitative Study

**DOI:** 10.3390/tropicalmed7100304

**Published:** 2022-10-16

**Authors:** Sophaphan Intahphuak, Thaworn Lorga, Worawan Tipwareerom

**Affiliations:** 1 School of Nursing, Mae Fah Luang University, Chiang Rai 57100, Thailand; 2 Faculty of Nursing, Chiang Rai Rajabhat University, Chiang Rai 57100, Thailand; 3 Faculty of Nursing, Naresuan University, Phitsanulok 65000, Thailand

**Keywords:** rational drug use, medicine prescribing, thailand primary healthcare, medicine use policy, nursing prescriber

## Abstract

Background: To address the problems related to drug resistance and medication safety, the rational drug use (RDU) policy has been implemented in Thailand since 2014. Theoretically, the policy was supposed to bring drastic changes to the way clinicians prescribe medications and its impacts on clinical practice, however, it has not yet been investigated. The study aimed to describe the experience of community health nurses with regard to the impact of RDU policy implementation on their practices. Methods: Focus group interviews and in-depth interviews with community nurses were conducted. Thematic analysis was performed. Results: Five themes emerged from the analysis, namely, (1) a welcome opportunity, (2) RDU as the quality of healthcare, (3) multidisciplinary collaboration, (4) reinventing productive interactions between nurses and patients, and (5) challenges over control of medications prescribed or purchased elsewhere. Conclusions: Implementing RDU in primary care provides opportunities for protecting individual patients and public health as well as safeguarding against professional prescription error. This can be made possible by adopting a systemic approach to changes. Additional educational and organizational support will optimize health professionals’ contribution to the implementation and hence optimal outcomes of this important policy.

## 1. Introduction

Medicine is one of the critical factors that contribute to health system efficiency. The right medicine leads to decreased morbidity, severity, and mortality rate. On the other hand, irrational drug use, especially antibiotics, is a serious public health problem that is harming patients with unnecessary side effects, antimicrobial resistance, and poor health outcomes [1]. Antibiotic drug resistance is a critical “One Health” issue affecting humans, animals, and the environment and requires a united multi-sectoral collaboration to reduce the problem [2]. Promoting RDU in healthcare professionals is needed for the responsible use of antibiotics to slow down the development of antibiotic resistance [1].

In Thailand, preliminary research suggests that there are approximately 88,000 cases of antimicrobial-resistant bacterial infections in humans each year, of which 38,000 result in death; this is equivalent to an economic impact of THB 42 billion [3]. The irrational use of antibiotics is not only found in hospitals but it also in the community. There are many factors that lead to the irrational use of medicine, including the lack of prescriber knowledge, prescriber habits, and lack of clinical guidelines and supervision [4]. Most patients are expected to be prescribed antibiotics when visiting doctor’s offices. Therefore, the prescription of antibiotics is used as a tool to satisfy patients to guarantee their revisit in the future [5]. Moreover, Thai people can obtain antibiotics from various sources such as hospitals, community primary care centers, pharmacies, and grocery stores [6].

Thailand’s national strategic plan on antimicrobial resistance 2017–2021 was endorsed by the Thai Cabinet in 2016. It aimed to decrease antimicrobial morbidity by 50% and increase public knowledge about antibiotics and antimicrobial resistance (AMR) awareness by 20% by 2021. The plan consisted of six strategies: (1) antimicrobial resistance surveillance system using a “One Health” approach, (2) regulation of antimicrobial distribution, (3) infection prevention and control and antimicrobial stewardship in humans, (4) AMR prevention and control and antimicrobial stewardship in agriculture and animals, (5) public knowledge on antimicrobials and awareness of appropriate use of antimicrobials, and (6) governance mechanism to develop and sustain antimicrobial resistance related actions [7].

As noted in Clinical Infectious Diseases, “Nurses are antibiotic first responders, central communicators, coordinators of care, as well as 24-h monitors of patient status, safety and response to antibiotic therapy” [8]. In Thailand, most community nurse practitioners work for the community primary care center in the sub-district and generally take care of 10–30 patients per day [9]. They are responsible for health assessment, diagnosis of common health problems, prescribing and administering, referring the patient to the appropriate hospital depending on the severity of illness, and continuity of care for chronic patients in the community following the Thailand Nursing and Midwifery Council medical lists and scope of practice guideline [10].

Nurses who work in the community primary care centers play an important role in healthcare services due to the shortage of physicians. There were no available posts for physicians, pharmacists, or assistant pharmacists in the centers. Therefore, disease screening, monitoring, treatment, and prescribing are managed by registered nurses or community nurse practitioners. Notably, nurse practitioners are increasing as prescribers of antibiotics in primary care centers and other settings, making them well-positioned to combat antibiotic resistance through appropriate antibiotic prescribing and patient counseling. Antimicrobial stewardship is an integral component of patient safety. Nurses can make significant contributions to practice, educate, research, and make policy efforts to reduce antibiotic resistance [11]. The study aimed to describe the experience of community nurses with regard to the impact of RDU policy implementation on their practices.

## 2. Materials and Methods

### 2.1. Study Design

A descriptive qualitative design based on a thematic analysis was used for data collection and analysis.

### 2.2. Study Setting

This study was conducted in the Wiang Pa Pao District, Chiang Rai Province (located in the northern part of Thailand). The locations are approximately 86 km from Chiang Rai Province, Thailand. The health centers were located in ten sub-districts responsible for a total population of 49,648. The top three infectious diseases among the people living in the selected district were (1) diarrhea, (2) respiratory tract infection, and (3) skin infection [12].

### 2.3. Data Collection and Tool Development

A semi-structured interview guide with open-ended questions was used to facilitate reflection. Nine questions were developed to collect data on the experiences regarding the RDU policy implementation. The examples of questions were:

(1) How do you feel about the RDU policy?
(2) What have you learned from RDU policy?
(3) Is there any problem during the RDU policy implementation?

Credibility was enhanced by informal member checking at the end of each focus group and in-depth interviews. A clear audit trail showed that all findings were derived directly from the data to ensure conformability and fair representation of data. Furthermore, rigorous translation checks increased dependability.

### 2.4. Recruitment of Participants

Participants were community health nurses who worked in Wiang Pa Pao District. The inclusion criteria in the study were: (i) at least 1 year of nursing experience in a primary care center, (ii) prescribing experience, and (iii) willingness to provide information. Participants were excluded if they were not willing to give their work information. The invitation letters were sent to the community primary care centers. If the community health nurses were willing to participate in the research, they were asked to directly contact the researchers. Twelve community nurses from ten community primary care centers consented to participate. According to homogeneity, twelve participants were separated into two focus group interviews by nursing experience (under 10 years and above). Initially, two focus group interviews were conducted in December 2019 by the first author. None of the participants were known to the primary author prior to the focus group interviews.

### 2.5. Data Collection Process

Participants completed a demographic survey (e.g., age, nursing experience in primary medical care, and short course training in nurse practitioner), followed by participation in the focus group interviews to explore the RDU policy in primary care centers, to prescribe antibiotics patterns, and to consider benefits and barriers of RDU in antibiotics used. Each focus group interview ranged from 1 to 1.5 h and was conducted in a private conference room of the district health office. Then, researchers selected two participants, not only allowing for more insightful information but also positive and negative reflections for the next interview. Then, the Snowball methodology was used to recruit in-depth interview participants. Interviews were conducted with the nurse in focus group interviews (*n* = 3) and new interviewees (*n* = 2). The in-depth interviews, lasting an average of 1 to 1.5 h, were performed in January and February 2020 by the first and second authors at the participant’s workplace, to explore RDU policy in the workplace, RDU implementation, and impact and barriers to RDU policy. All focus group and in-depth interviews were audio-recorded and transcribed verbatim by the first author.

### 2.6. Data Analysis

Thematic analysis was used to derive the key themes from the data [13,14]. All interviews were analyzed individually and then collectively using an analytical framework developed by the team of authors. Themes from the data were initially identified by the first and second authors and subsequently verified by all authors for coding consistency, the emergence of main themes, and extraction of statements to support the themes that were selected to present the findings.

### 2.7. Ethical Issues

The study concepts were approved by the Chiang Rai Provincial Health Office Ethics Committee on Human Research, Thailand (reference no. CRPPHO 8/2562).

## 3. Results

Twelve participants were recruited (11 in the focus group and 5 in-depth interviews) as indicated in Table 1. All of them were female and worked in community primary care centers. Most participants worked as nurse practitioners. Their ages were between 26–58 years old (mean = 38.75, SD = 11.41). The highest work experience was 34 years and the lowest was 4 years (mean = 15.50, SD = 11.78).

### 3.1. RDU Is a Welcome Opportunity

Since the beginning of the RDU policy implementation, every community primary care center in Thailand is required to comply with prescription guidelines and protocols. Drugs that were previously prescribed at the primary care center are no longer prescribed. These medications, including certain antibiotics, steroid drugs, and paracetamol injections, are prescribed only by doctors at community hospitals and higher-level healthcare settings. Despite drastic changes in prescriptive authority, all the nurses and public health practitioners in this study have witnessed the positive side of these changes. They look at such changes as a welcome opportunity because it helps prevent prescription error. In addition, having guidelines made them confident in their treatment and management decisions.

*“I feel good about RDU. I think this is good. It’s wonderful. This is because we follow the (prescribing) criteria and if anything goes wrong, we can always say that we have followed the written standard. The standards will protect us.”*

(FG#1-N6).

*“I don’t feel frustrated at all. I mean our current (nursing) scope of prescribing practice is just fine. We prescribe within our scope of practice, and we are safe.”*

(FG#2-N10).

### 3.2. Rational Drug Use as Quality of Healthcare

Rational drug use is part of the key performance index (KPI) for primary care award (PCA), also called starred sub-district health promoting hospital (Starred SDHs). The criteria standard on RDU and safety are (1) guidelines on antibiotics used to treat respiratory tract infections, acute diarrhea, and wounds from accidents, and (2) antibiotics used to treat respiratory tract infections and diarrhea should not be more than 20 percent which can be evaluated from the Health Data Center (HDC) report. These criteria were used to monitor the operational system of the community primary care center for the RDU from the Thai Ministry of Public Health (MOPH).

*“When the overuse of antibiotics is found, the pop-up ring will alarm in the computer program. Therefore, we are careful to prescribe the antibiotics, we recheck again and again if the patients’ symptoms need antibiotics.”*

(QI#5-N11).

In contrast, the nurses with less than ten years of experience were still hesitant about RDU since there was no evidence supporting the policy implementation.

*“Are the patients really safe? Do drug resistant infections really decrease?”*

(QI#4-N9).

*“There is no evidence or any reports about RDU efficacy.”*

(QI#5-N11).

### 3.3. RDU Requires Multidisciplinary Collaboration

The primary care and treatments were mainly performed by nurse practitioners or registered nurses who work in the primary care center. Nurses could consult cases with doctors and pharmacists via LINE groups. The district hospital assigns one doctor responsible for consulting 2–3 primary care centers. Most participants felt more confident about disease diagnosis and antibiotic prescription when they were working under a doctor’s monitoring. The doctor consultation system made them feel safe and decreased medication errors.

*“It can increase our confidence for the treatment and drug prescribing. We can direct messages and video calls via LINE or phone to consult a doctor, and he will give suggestions and explanations about the disease.”*

(QI#4-N9).

*“It looks like we have the backup from a doctor. I feel safe if I consult the case with the doctor.”*

(QI#5-N11).

### 3.4. RDU Reinvents Productive Interactions between Nurses and Patients

Rational drug use policy opened an opportunity for a nursing profession in health promotion. Health education was mainly used to explain infectious disease protection and rational antibiotic use. Most participants had experiences with patients requesting medication. After the RDU policy implementation, they felt comfortable communicating with their patients.

*“It is a good opportunity for nurses to promote people’s health. Community nurse’s obligation should provide health prevention and health promotion more than treatment.”*

(QI#4-N9).

*“Our primary care center worked on RDU both in the primary care center and the outreach. We went to every village that we were responsible for (10 villages). We gave the knowledge about infectious diseases, protection, and rational antibiotics use.”*

(QI#3-N2).

### 3.5. Challenges over Control of Medications Prescribed or Purchased Elsewhere

Some patients with mild symptoms (e.g., headache, common cold, toothache, stomach ache, and muscle strain) can access medicines from pharmacies and community groceries where medicines including nonsteroidal anti-inflammatory drugs (NSAIDs), antibiotics, and even steroids are available without prescriptions. As a result, irrational drug use was found in the community, especially in adults and elderly people. It is one challenge of the public health problem for health professionals to address.

*“I found NSAIDs, antibiotics and “Yaa chud” (which include analgesic, NSAIDs, and steroids in one set and the patient will take them all at one time) available which were prohibited in community groceries. There is no strict penalty and measure for them except they had to pay a fine that did not come too much.”*

(FG#1-N)

*“The advertisement from radio, information from other patients, and word of mouth in the community are the most influence on self-medication.”*

(QI#5-N11)

One participant has seen an irrational drug prescription from a doctor.

*“Nurses always strictly prescribed medicine following the RDU guideline, while some doctors prescribe the medicine depending on patient conditions. I had seen a doctor prescribing overuse of medicine to a hill tribe patient who lived in a remote area. I have ever mentioned the problem, but the doctor still prescribes. I thought the doctor might have his own reason to do that.”*

(QI#3-N2).

## 4. Discussion

This study found that RDU implementation in community health settings (e.g., antibiotics smart use guideline, standard operation procedures for primary care, medicine lists for nurses in primary care centers, interprofessional work, KPI for antibiotics use, and primary care awards, health education about disease and medicine) are warranted. The activities were conducted by following the WHO’s twelve core interventions to promote the rational use of medicine that includes clinical guidelines, an essential medicines list based on treatments of choice, drugs and therapeutics committees in districts and hospitals, supervision audit and feedback, independent information on medicines, public education about medicines, and appropriate and enforced regulation [15].

### 4.1. RDU Is a Welcome Opportunity

Most of the participants in this study were nurse practitioners. They demonstrated their duties by following the RDU policy. Nurse practitioners are important health professionals who influence new policies and positive changes in healthcare [16]. A positive attitude toward RDU policy was found in the study. The attitude about change in practices (as part of a policy change) is key to health professionals’ acceptance and adherence to change guidelines. Following RDU policy in this study attributed to the removal of prescription error risk, feeling protected by the proposed change, having readily available tools for decision making, having well-found knowledge and understanding, having good relationships interprofessionally, performing a health promotion role, and having established adequate confidence of treatment and prescribing. The good opportunity from RDU in this study is similar to a previous study which revealed that diagnostic and treatment knowledge, patient consultation, and interprofessional work are opportunities for implementing strategy [17].

Prescribing practice is a complex task that requires many skills such as diagnostic, clinical pharmacology, communication, and decision-making based on judgments of potential benefits and risks to patients [18]. The RDU-prescribing practice in this study conforms to results from a study in South Africa, which found that the professional nurses at primary healthcare clinics had a good understanding and prescribed by following standard treatment guidelines and the essential medicines list [19]. The positive attitude toward RDU and an advantage in patient outcomes are influenced by the rational drug prescribing of health professionals [20,21].

### 4.2. RDU Used as Quality of Healthcare

The responsible use of medicines is an essential element in achieving quality care for patients and the community [22]. The first and most critical step of quality improvement is measuring the quality. Quality indicators can be used to promote quality improvement activities, measure process indicators, and compare the quality of care provided by healthcare professionals in primary care and hospitals [22,23]. The hospitals need to encourage and promote the systematic monitoring of antibiotic use through the institution of programs [24]. In this study, the RDU, especially antibiotics prescription, was used as KPI for primary care awards. The award was one strategy of the MOPH to motivate and maintain the RDU. The criterion for starred SDHs standard is an effective strategy for the development of primary care prescribing in the community [25]. In addition, evidence shows that implementing the RDU campaign could effectively improve rational antibiotic prescribing in primary care and hospital in both short- and long-term maintenance [26,27], whereas the reward plays a motivational role to support and urge workers to produce a good performance and high-quality work [28,29].

The participants mentioned that data on the efficacy of RDU policy still has not been reported. They will have more confidence in antibiotic prescribing if the efficiency report is available. According to previous reports, nurses and midwives always use clinical effectiveness as criteria for their prescribing [30]. Rational prescribing decisions are often based on evidence. The clinical trial data concerning medicines in common usage might be expected to provide sufficient evidence to support the prescribing decisions [18]. The five years of Thailand’s national strategic plan on antimicrobial resistance have not been fully reported. There is only a report on decreasing the level of and trend in antibiotic prescribing rates during the years 2017–2019 from the RDU Service Plan [31]. Reports should evaluate not only the decrease in antimicrobial consumption but also the incidence and prevalence of AMR compared with the antibiotics prescription or consumption rate.

### 4.3. RDU Requires Multidisciplinary Collaboration

This study found that nurses felt free to communicate with physicians and pharmacists because they were always willing to help and available anytime for consultations via a social media application. The interprofessional collaborative facilitators in this study are similar to several studies that found that good collaborative relationships among healthcare team members are key for nurse practitioners in their ability to make optimal patient care decisions [32], effectively and safely prescribe [33], and maintaining quality patient care [34]. RDU implementation established a scope of practice, responsibility, and essential duty of community health nursing, and created trust among health professionals [35]. Trust between doctors and nurses was shown to be necessary for effective RDU integration; without trust, the nurses will not prescribe [36]. The step-by-step collaboration development between nurse practitioners and medical practitioners, and the positive experiences of working collaboratively may be the strongest force to enhance and advance the collaboration [37]. This effective teamwork should be the criteria for a successful outcome.

### 4.4. RDU Reinvents Productive Interactions between Nurses and Patients

Community health nurses providing community health services include health promotion, disease and injury prevention, rehabilitation, managing and providing care, and follow-up across various settings [35]. The worthiest professional aspects of the nurse role include patient interactions, respect, and interprofessional collaboration [38].

This study reveals that the RDU policy reinvents the productive relationship between patients and community health professionals. Effective communication is one of the main competencies of community health nurses [35]. The increased value of community health nurses could implement via RDU policy by using effective health education. Nurses should give drug information to their patients such as name, clinical use, frequency, side effect(s), storage, and expiration date. In the first phase, time and effort may be required for healthy communication with individuals, families, groups, and communities. Eventually, the impact of health information on patient outcomes will include a good relationship, trust, positive attitude, and understanding of RDU policy.

### 4.5. Challenges over Control of Medications Prescribed or Purchased Elsewhere

Irrational drug use, such as prescribed drug overuse in hospitals, the selling of prohibited medicines in pharmacies and community groceries, and self-medication were reported in this study. The prescribing practices elsewhere (e.g., pharmacies, groceries, and private clinics) do not follow the RDU guidelines adopted by the government healthcare facilities which are beyond the nurse’s control. Some medicines (such as antibiotics and steroids) are not available in a primary care center, but a patient could buy them from community groceries. This finding is similar to the results observed in several studies, such as steroid self-medication or “Yaa chud” (in Thai), which showed the important problem. The selling of “Yaa Chud” in Thailand is illegal but the loosely regulated drug laws mean that most sellers (such as community groceries and small pharmacies) of “Yaa Chud” are rarely prosecuted by the Food and Drug Administration Thailand [39]. The challenges, however, lie ahead. Irrational drug use from other sectors may impact RDU policy implementation. Self-medication is an important factor that leads to irrational drug use. Most patients would like relief from their symptoms as soon as possible, so they find the strongest and the best medicine even if it has serious side effects. Therefore, supporting people to access, understand, evaluate, and apply drug and health information appropriately through drug literacy is required [6].

## 5. Conclusions

The success of RDU policy implementation in community healthcare centers involves many factors, such as an open mind for critical opportunities, improving and developing multidisciplinary collaboration, productive interaction between nurse and patient, and a change in perspective from “beyond the control” to just challenges, which lead to increased RDU implementation quality and support the “One Health” approach to battle antibiotic resistance. Nurse practitioner courses in Thailand should include pharmacology knowledge and an RDU program for nurses. The lessons learned from the study include that RDU implementation should create a good attitude, clinical guidelines, consultations with physicians and pharmacists, and interprofessional collaboration, and increase the nurse’s value by providing health education which is an important role of the community nurse.

## Figures and Tables

**Table 1:** Demographic characteristics of the participants.

Nurse	Focus Group	In-Depth Interview	Official Title	Age (Year)	Professional Experience (Year)
N1	FG#1		Nurse Practitioner	58	34
N2	FG#1	QI#3	Nurse Practitioner	52	30
N3	FG#1		Nurse Practitioner	52	28
N4	FG#1		Nurse Practitioner	43	21
N5	FG#1		Nurse Practitioner	47	24
N6	FG#1	QI#1	Nurse Practitioner	41	20
N7	FG#2		Nurse Practitioner	29	4
N8	FG#2		Nurse Practitioner	30	8
N9	FG#2	QI#4	Nurse Practitioner	30	5
N10	FG#2		Nurse Practitioner	26	4
N11	FG#2	QI#5	Registered Nurse	29	4
N12		QI#2	Registered Nurse	28	4
				mean = 38.75 SD = 11.42	mean = 15.50 SD = 11.78

Note. FG = focus group; QI = in-depth interview.

## Data Availability

The datasets used and/or analyzed during the study are available from the corresponding author on special request.
